# A little bit is better than nothing: the incomplete parthenogenesis of salamanders, frogs and fish

**DOI:** 10.1186/1741-7007-8-78

**Published:** 2010-08-03

**Authors:** Kathrin P Lampert, Manfred Schartl

**Affiliations:** 1 Animal Evolutionary Ecology and Biodiversity, University of Bochum, Universitätsstrasse 150, 44780 Bochum, Germany; 2 Physiological Chemistry I, Biocenter, University of Würzburg, Am Hubland, 97074 Würzburg, Germany

## Abstract

A re-examination of the mitochondrial genomes of unisexual salamander lineages, published in *BMC Evolutionary Biology*, shows them to be the oldest unisexual vertebrates known, having been around for 5 million years. This presents a challenge to the prediction that lack of genetic recombination is a fast track to extinction.

See research article http://www.biomedcentral.com/1471-2148/10/238

## Commentary

About 90 species of vertebrates have been discovered that are strictly unisexual, and all of them are fish, amphibians or reptiles [1]. Being all females, every individual produces offspring, and thus the population will grow much faster than any competing bisexual species that has to generate males. In bisexual species, males exist only for the purpose of donating 50% of the freshly recombined genetic material and do not produce offspring, and this burden has been termed the 'twofold cost of sex' by John Maynard Smith [2,3]. Considering the reproductive advantage for a unisexual species, one would expect that many more unisexual vertebrate species should exist. There are, however, also disadvantages to the absence of genetic recombination. One major problem is that without meiotic crossovers, deleterious mutations cannot be purged and thus they accumulate in the genome - a process known as Muller's ratchet [4]. A second problem is that the genetic uniformity of the offspring leads to a much lower genetic diversity, which is likely to make it much more difficult to adapt, for example, to changing environments or to parasites; consequently, asexual species should be slow to evolve [3,5]. These two disadvantages are predicted to strongly outweigh the reproductive advantage, with unisexual lineages being predicted to go extinct within a short time (10^4 ^to 10^5 ^generations [6]), explaining why they are so rare.

Like all good theories, this explanation for the rarity of unisexual vertebrate species can be tested. In a study published recently in *BMC Evolutionary Biology*, Bi and Bogart [7] set out to examine the evolutionary age of species of salamanders of the genus *Ambystoma*. This group is known as the 'mole salamanders' and contains about 30 bisexual species distributed from the Central Valley of Mexico to Alaska and Labrador, along with several unisexual biotypes (a biotype is a group of individuals with the same genotype), which are abundant in the Great Lakes region of North America. Astonishingly, the nuclear DNA content of the unisexual biotypes ranges from diploid to pentaploid, and their nuclear genomes are apparently combinations of haploid genomes or multiples thereof from four bisexual species: *Ambystoma laterale*, *A. jeffersonianum*, *A. texanum *and *A. tigrinum*. For instance, the triploid unisexual biotype LLT has two copies of the *A. laterale *genome (L) and one copy of the *A. texanum *genome (T). Of the more than 20 different unisexual biotypes identified so far, all have at least one L genome, but everything else is variable. In contrast, the mitochondrial genomes (mtDNA) of all *Ambystoma *unisexuals are very similar to that of another species, *A. barbouri*. This supports the hypothesis that, like all other unisexual vertebrates, the unisexual salamanders are of hybrid origin, and that *A. barbouri *was the maternal species involved in the hybridization. The Kentucky genotypes of *A. barbouri *seem to be most closely related to the unisexual lineages.

Bi and Bogart have analyzed mtDNA sequences, including that for cytochrome b (*cytb*) and non-coding control regions, from 46 individuals of 9 unisexual biotypes and one *A. laterale *individual as an outgroup. They also constructed a phylogeny from the complete mitochondrial genomes of one of the unisexual biotypes, two *A. barbouri *individuals, one *A. texanum *individual (sequenced in this study) and 13 other amphibians. Their data support a monophyletic and ancient origin of the unisexuals in the *Ambystoma *complex. No mitochondrial genotypes were shared between the unisexuals and *A. barbouri*; instead, the sequence divergence in *cytb *between unisexuals and *A. barbouri *was 5.16%. From this, the split between *A. barbouri *and the unisexual lineages was estimated to have occurred approximately 5 million years ago, based on conservative estimates of the mutation rate in mitochondrial sequences. Bi and Bogart's age estimate is in good agreement with earlier studies, which suggested an age for the unisexuals of 2.4 to 3.9 million years [8]. This finding makes the unisexual *Ambystoma *salamanders the oldest clonally reproducing vertebrates known.

The unexpectedly old age of the unisexual salamanders is intriguing - and they are not alone. Other unisexual vertebrate species, although not reaching the antiquity of *Ambystoma*, are also much older than would be predicted on theoretical grounds. The Amazon Molly, *Poecilia formosa*, is a small all-female live-bearing fish species that occurs in fresh water in the northeastern lowlands of Mexico up to the Rio Grande. Its age has been calculated on the basis of mitochondrial and nuclear sequences to be 280,000 years and approximately 800,000 generations [9]. In the genus *Poeciliopsis*, which also belongs to the same family of live-bearing fishes, several unisexual biotypes exist in the rivers of northwest Mexico. Calculations suggest that these biotypes are more than 60,000 years old, equivalent to up to 200,000 generations [10]. Both fish are well above the predicted upper survival limit of 10^5 ^generations and show no signs of decline. They are successful colonizers and very abundant.

## How do they do it?

The question then arises: are the hypotheses about the consequences of the absence of recombination wrong, or are the age estimates? Most probably, both are correct. The solution to this paradox comes from the fact that many unisexual vertebrates have specialized ways to circumvent the lack of meiotic recombination in their nuclear genome (see [1]).

Although all unisexual female reproduction is often loosely called parthenogenesis (reproduction in the absence of fertilization of the egg), true parthenogenesis is much more restricted. Defined as the production of offspring by virgin females in the total absence of males, true parthenogenesis results in genetically identical clonal populations. In this exclusivity, true parthenoforms of vertebrates are only found in unisexual lizards and the single unisexual snake species known to date. The unisexual fish and amphibians, in contrast, reproduce by variations of parthenogenesis, which are incomplete and genetically leaky compared with true parthenogenesis.

**Figure 1:**
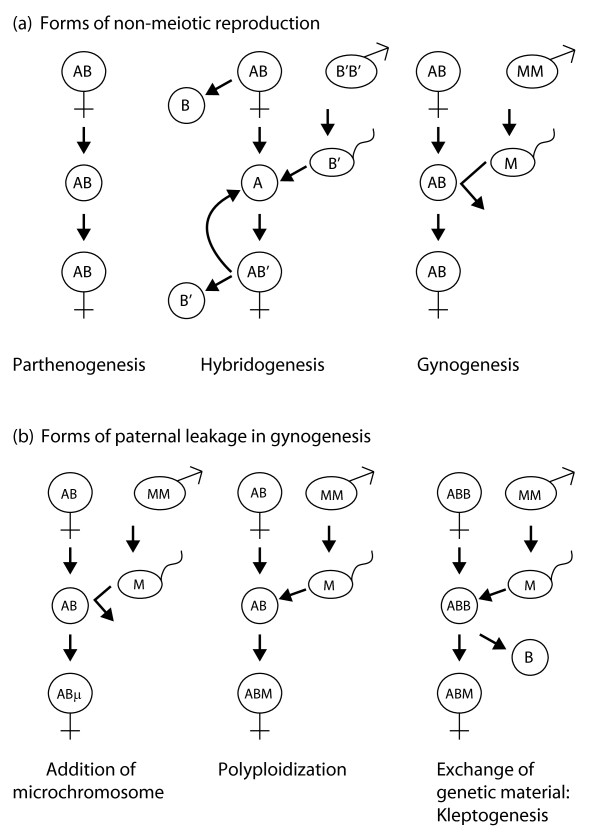
**Variations on parthenogenesis**. **(a) **Schematic representations of forms of non-meiotic reproduction. Starting genomes in this illustration are shown as maternal diploid (AB) or triploid (ABB) and paternal diploid (MM). True parthenogenesis does not require any intervention by sperm; diploid oocytes develop directly into diploid offspring of identical genotype to the mother. Hybridogenesis and gynogenesis involve the intervention of sperm. In hybridogenesis, a haploid oocyte is produced without meiosis and is fertilized by a sperm, which contributes its genome (M) to the offspring. However, the M genome is lost when oocytes are produced in the next generation, so the oocyte always contains an unchanged maternal genome. In gynogenesis, stimulation by sperm is required for the oocyte to develop into an embryo, but the sperm does not contribute any of its genetic material to the offspring. **(b) **Ways in which 'paternal' DNA can leak into gynogens. From left to right: small pieces of chromosomes from the sperm can be retained in the oocyte as microchromosomes (μ); a full sperm M haploid genome can be added, leading to polyploidization; all or part of a maternal genome can be replaced by the sperm M genome (kleptogenesis).

One variation of parthenogenesis is gynogenesis, in which meiotic reduction does not occur during oogenesis, but sperm is needed to trigger the onset of embryonic development. The sperm is provided by a male of a related species, but the male genetic material is usually excluded and does not contribute to the genotype of the offspring. Very rarely the exclusion mechanism fails, and either small bits of 'paternal' DNA are included in the oocyte in the form of additional B chromosomes (Figure 1, microchromosomes), or the oocyte is fertilized, leading to an increase in ploidy in the offspring (Figure 1, triploidy). An extension of gynogenesis is the reproductive strategy of kleptogenesis, in which part or even all of a maternal genome is more or less frequently exchanged for paternal genetic material [8]. This is the typical reproductive mode of the unisexual *Ambystoma*. If, for instance, a triploid female of genome type LLJ (laterale/laterale/jeffersonianum) gets its trigger for kleptogenesis from an *A. texanum *male, the resulting offspring could consist of both LLJ individuals (no genetic exchange occurs) and LTJ individuals (genetic exchange has occurred). In the latter case, one L genome has been replaced with a T genome derived from the 'father'. Kleptogenesis therefore allows the acquisition of highly adapted genetic material by the otherwise non-recombined unisexual genome.

A third form of leaky parthenogenesis found in some vertebrates is hybridogenesis. In this mode of reproduction, haploid oocytes are produced without meiosis. The oocytes are fertilized by sperm from a closely related bisexual species but the male genetic material is only present for a single generation; it is excluded during oocyte production and consequently is not passed on to the next generation, and so the oocytes always exclusively contain a maternal genome.

These leaky forms of parthenogenesis very rarely, or not so rarely, allow the inclusion of paternal genetic material in the oocyte. The consequence is a constitutive or occasional addition of 'fresh' genetic material that can slow down the degeneration process of Muller's ratchet and increase genetic diversity.

This then raises the question: why are unisexual vertebrate species so rare if they have found ways to decrease the negative impact of having no meiosis but simultaneously enjoy the advantage of enhanced population growth? An attractive hypothesis is that unisexuals are so rare not because they are under considerable disadvantage compared to their bisexual competitors, but because the genomic conditions under which they can arise are extremely rare [11]. Evidence for this comes from a study on the gynogenetic Amazon Molly, *P. formosana *[12]. The diploid genome of the asexual biotype is composed of one copy from its maternal ancestor, *P. mexicana*, and one copy from its paternal ancestor, *P. latipinna*. Both these species still live together at some places in northeast Mexico, and there has been ample opportunity for hybridization. In the laboratory, hybrids between these two species, carrying both genomes, are easily produced, but they are not the expected gynogens of the *P. formosa *type. *P. formosa*, like the mole salamanders, has been found to be monophyletic and of rather ancient origin. All this shows that the hybrid composition of the genome is not on its own sufficient to initiate asexuality. Only certain combinations of individual genomes from the genome pools of both species, and possibly additional mutations in the hybrid, can bring about the switch from bisexual to unisexual reproduction.

The paper by Bi and Bogart [7] is a crucial cornerstone to our understanding of the biology of unisexual vertebrates and the evolution of asexuality versus sexuality in general. The reproductive mode of kleptogenesis in the mole salamanders and the other mechanisms of incomplete parthenogenesis in unisexual fish and other amphibians have obviously ensured their long-term survival, and tell us that 'a little bit of sex' gives these organisms the best of both worlds - just enough genetic variation in addition to the mutations that generate new genotypes also in asexuals, plus the superior mode of propagation in the absence of males.
